# Lobelin

**Published:** 1910-03

**Authors:** George L. Servoss

**Affiliations:** Fairview, Nevada


For Texas Medical Journal.

### Lobelin.

BY GEORGE L. SERVOSS, M. D., FAIRVIEW, NEVADA.

In close connection with emetine and sanguinarine, which have been discussed to some extent in the *Journal,* is lobelin, a concentration from *Lobelia inflata.* According to Lloyd and Felter, lobelin is an extremely active emetic, so active in fact that it is impossible to administer a poisonous dose. They say that death in a very prostrated case might result from excessive vomiting, but not from the direct action of the drug. When the entire drug, lobelia, is chewed it causes an acrid, pungent sensation, with slight nausea, warmth and distension of the esophagus and stomach, stimulation of the salivary and buccal glands, followed by epigastric depression with profound nausea, and with large doses, severe and thorough emesis. The authorities say that this depression is transitory and is immediately followed by a sense of extreme satisfaction and repose. The mental powers are rendered very acute by the action of this drug. Large doses depress, while small doses stimulate, the circulation. It increases the bronchial secretions and if it does not produce emesis, purging follows. It produces death by paralyzing the respiration. Like the entire drug, lobelin is nauseant, emetic, expectorant, relaxant, antispasmodic, diaphoretic, sialagog, sedative, and occasionally cathartic, diuretic and astringent. It has no narcotic action. While it produces temporary depression, it is preferred to all other emetics, because of its beneficial after effects. Combined with ipecac, it is said to be safer and more effective.

It is recommended in the forming stages of fevers, with sluggishness and heavily-coated tongue; in chronic diseases with atony; as an emetic in small doses, in warm water, frequently repeated. Nauseant doses in chorea, tetanus, worm fits of children, hysteric and the convulsions of infants, and in eleptiform and other convulsions. It is said to be the remedy par excellence in rigid os uteri, relaxing the perineum as well, and is recommended where the os is thick and doughy, although gelsemium acts better for the thin, knife-edged os. It is also suggested in intestinal obstruction, strangulated hernia, intussusception, and fecal impaction; the spasm of asthma, croup, whooping cough, strychnine poisoning, and as a sympathetic stimulant to improve the innervation of the pneumogastric and sympathetics. It is useful in indigestion, dyspepsia, gastric sick headache, qualmishness and nausea, intestinal atony and habitual constipation, for costiveness when combined with podophyllin. It increases peristalsis and small doses give relief in infantile colic. It is one of the best, if not the best, remedies for markedly slow pulse-wave, with precordial oppression, thoracic pain, difficult breathing, soreness in the chest, nausea, tongue with heavily-coated base and fullness of the tissues; cardiac congestion, and is of great value in respiratory affections. It is *the* drug for angina pectoris, cardiac neuralgia and pulmonary apoplexy. It gives relief in the dry, hard barking of chronic catarrhs and in coughs and colds, and is useful in all respiratory irritations with congestion or oppression, full oppressed or small feeble pulse with oppression in the chest, accumulated secretions and loud mucous rales; in the retrocession of eruptions, scarlatina and measles with tardy eruptions, rhus poisoning, locally in numerous superficial inflammatory conditions; in erysipelas.

The specific indications are full, labored, doughy pulse, the blood moving with difficulty; heavy, sore, oppressive chest pains; angina pectoris, cardiac neuralgia, pulmonary apoplexy; accumulations of mucus in the bronchi; convulsive movements; rigidity of the muscles and os uteri with thick, doughy edges, rigid perineum or vaginal walls; nausea and sick headache and as an emetic when the tongue is heavily coated at the base.

Ellingwood considers lobelia as the best remedy acting upon the respiratory tract and says that it is a nerve depressant of great power, and recommends it as a specific in irritable, spasmodic and oppressed breathing; threatened spasm with exalted nerve action; high nerve tension with great restlessness and excitability, flushed face and contracted pupils; for eclampsia and the spasms of hydrophobia. He says that it affects children less than adults. He recommends lobelia in combination with capsicum.

Lobelia has been considered highly by the eclectic for a long time, it having held the first place with them for nearly a century, since first being introduced by Thompson, and the foregoing is a resume of their findings regarding the drug. The concentration, lobelin, has all of the activity of the entire drug.

There is an alkaloid, lobeline, derived from the plant, which Cushny says increases reflex irritability and accelerates respiration by stimulating the centers of the cord and medulla. The blood pressure first falls, but soon rises above normal. The respirations are rendered faster, deeper and stronger. He says that its action, in some respects, resembles that of nicotine. He limits the use of lobeline to nervous asthma.

Cornet says that lobeline sulphate is effective in the spasmodic cough of phthisis, and in the various forms of croup, and that it is also useful in chronic bronchitis, asthma, and the convulsive cough of pertussis.

While little is said of this drug in connection with its power to loosen the secretions in dry catarrhal affections it would seem to be one of the best remedies offered, in fact this seems to be the one specific indication for the use of lobelin.

The dose recommended is one-twelfth to one-half grain, always exhibited in hot water, this due to the tendency of the drug to pass through the bowel without going into solution. Smaller doses are recommended for costiveness in children.

Lobelia is a drug that has not received great consideration, excepting in the ranks of the eclectics, but from the foregoing it would appear to be a very valuable addition to the armamentarium of the practician and is worthy of more attention than has heretofore been given it. Lobelin, because of its purity and standard strength, is probably the best form in which the drug is offered. Like all other active principles, it is stable, of exact strength and affords of greater certainty of dosage, and is far more pleasant of administration than is any other form of the drug.

